# Bone Marrow Aspirate in Spine Surgery: Case Series and Review of the Literature

**DOI:** 10.7759/cureus.20309

**Published:** 2021-12-09

**Authors:** Thomas Noh, Hesham Zakaria, Lara Massie, Christian T Ogasawara, Gunnar A Lee, Mokbel Chedid

**Affiliations:** 1 Division of Neurological Surgery, University of Hawaii John A. Burns School of Medicine, Honolulu, USA; 2 Department of Neurological Surgery, Henry Ford Health System, Detroit, USA

**Keywords:** arthrodesis, case series, literature review, mesenchymal stem cells, bone marrow aspirate, spine surgery

## Abstract

Background

With the modernization of biotechnology, there has been a concerted effort to create novel biomaterials to promote arthrodesis for spine surgery. The novel use of the stem cells from bone marrow aspirate (BMA) to augment spine surgery is a burgeoning field because these cells are considered to be both osteoinductive and osteogenic. We sought to review the evidence behind the use of BMAs in spinal fusions and report the results of our own case series.

Methods

PubMed and EMBASE databases were searched for studies that investigated the use of stem cells for spine surgery. For our own case series, the medical records of 150 consecutive patients who underwent a lumbar spinal fusion with BMA were retrospectively reviewed for adverse events (AEs) for up to two years after surgery.

Results

In our case series, there were no AEs identified in 49% of our patients. Of the identified AEs, 61% were unrelated to the use of BMA (e.g., UTI and heart failure), with the remaining 39% likely unrelated to its use (e.g., back pain and anemia). There was a 92.8% arthrodesis rate with the use of BMA.

Conclusions

We reviewed the rationale, basic science, and clinical science for BMA usage in spine surgery and concluded that BMA is safe for use in spine surgery and is associated with a high rate of arthrodesis.

## Introduction

With the modernization of biotechnology, there has been a concerted effort to develop novel biomaterials that can be used to augment spine surgery. For example, the current gold standard to induce arthrodesis after spine surgery is iliac crest bone, because it has all the physiologic components required for bone growth; it is osteoconductive (provides a three-dimensional (3D) scaffold for new bone growth), osteoinductive (creates a microenvironment with growth factors that support bone growth), and osteogenic (has bone-forming cells that will lay the foundation for new bone). However, iliac crest bone has drawbacks; 15%-60% of patients complain of donor site pain after iliac crest harvest one to two years after surgery [1], up to 16.5% of patients have donor site pain worse than surgical site pain, 30% report donor site numbness, and 15% have difficulty walking because of donation [2]. A retrospective review of the National Surgical Quality Improvement Program (NSQIP) database has also shown that iliac crest bone grafting has been demonstrated to increase the rate of postoperative blood transfusion (11.6% versus 5.5%), extended operating time by an average of 22 minutes, and increased length of stay by 0.2 days [3]. However, rates of infection, return to the operating room, deep venous thrombosis, and readmission rates were not shown to be significantly increased within the iliac crest bone graft cohort.

Subsequently, new biomaterials are being developed to augment fusion by providing synthetic osteoconductive or osteoinductive materials. For example, allografts are man-made osteoconductive materials and have varying success in improving fusion [4]. The adverse events (AEs) associated with the use of allografts vary significantly in terms of severity. At the advent of this technology, cases of HIV, HCV, and other viral transmissions were reported, as well as transmission of bacterial infection [5]. However, these adverse events have essentially become nonexistent since improved safety and donor screening measures have been implemented. Recombinant human bone morphogenetic protein-2 (rhBMP-2) is a synthetic osteoinductive material that has known drawbacks, including high cost, no clinical guidelines, off-label usage, and a high rate of complications [6]. Osteolysis and subsidence are particularly serious adverse events associated with the use of rhBMP-2 secondary to known osteoclastogenic effects [7]. Cellular bone matrices, which are allografts with live stem cells embedded, are becoming increasingly common. However, the success of these constructs is influenced by industry bias, with nonindustry-funded studies showing a lower fusion rate [8].

Despite these novel advances, pseudoarthrosis is still common, occurring in 13%-41.4% of patients [9]. Synthetic material may be insufficient to overcome patient-derived factors that can affect bone healing, such as age, diabetes mellitus, smoking, autoimmune diseases (e.g., rheumatoid arthritis and lupus), chronic kidney disease, parathyroid disease, adrenal disease, malignancy, and osteoporosis/osteopenia [10]. Synthetic materials rely on the patient’s own cells to stimulate or grow new osteocytes to lay down new bone and thus by themselves are insufficient for arthrodesis because they are not osteogenic.

Bone marrow aspirate (BMA) has been used to overcome some of these deficiencies. The active component in BMA is the mesenchymal stem cell (MSC). MSCs are a small population of aspirates that is theoretically well poised to create a regenerative environment. The goal of this review is to discuss the physiology of BMA and MSCs, the basic science research behind their application, their use to augment arthrodesis after spinal fusion, the clinical research published, and our own results with the use of BMA.

## Materials and methods

After IRB approval (IRB# 9251), we reviewed the medical records of 150 consecutive patients who underwent a lumbar spinal fusion with the use of BMA from 2008 to 2011. We performed a retrospective chart review for any adverse events (AEs) that occurred for up to two years postoperatively. These events were categorized as related, possibly related, or unrelated to the use of the BMA. The specific features of the AEs included the timing of the AE in relation to surgery, whether the AE was expected related to the type of surgery (i.e., back pain, muscle spasm, leg pain, wound infection, durotomy, and anemia) versus unexpected (i.e., oral candidiasis, cardiogenic shock, deep vein thrombosis (DVT), neck pain, and adrenal insufficiency), whether and how the AE was treated, whether the AE resolved or was ongoing, and whether the AE was related to the use of BMA. AE severity was ranked as mild (i.e., pain responsive to medical treatment and anemia responsive to transfusion), moderate (i.e., ileus, hematoma, and post-laminectomy syndrome), severe (i.e., pneumonia requiring prolonged hospitalization and pulmonary embolism (PE) requiring anticoagulation), or serious (i.e., cardiogenic shock and failure to thrive).

Intraoperatively, BMA is collected by a previously standardized protocol [11]. Briefly, a Jamshidi needle is placed into the posterior superior iliac crest through the same incision used for lumbar surgery. The stylet is removed, and two 30 cc syringes preloaded with 1 mL of heparin (1,000 U/mL) are used to collect a total of 60 cc BMA, which is then concentrated via the commercially available CellPoint™ (ISTO Technologies Inc., Saint Louis, MO, USA) product. The concentrated BMA (cBMA) is combined with autograft, demineralized bone matrix (DBM), and allograft and then placed into interbody cages and posteriorly along the lateral gutters.

## Results

Characterization of adverse events

Of the 150 patients, 74 (49%) reported no AEs related to their treatment. The remaining 76 patients (51%) reported 124 AEs. Overall, AEs were identified 174 days after surgery. Regarding severity, 15% of the AEs (n = 18) were mild, 52% (n = 64) were moderate, 21% (n = 26) were severe, and 13% (n = 16) were serious. For the types of AEs, 28% (n = 35) were back pain only, 15% (n = 19) were radiculopathy only, and 6% (n = 7) were for both back pain and radiculopathy. Ten (8%) patients had wound infection, and nine (7%) had an infection not at the surgical site (i.e., UTI/pneumonia). The list of all types of AEs can be found in Table 1. Back pain, radiculopathy, or both accounted for 49% (n = 61) of AEs. Ten (8%) wound infections and nine (7%) infections at another site were documented. Other AEs included six deep venous thromboses, five ileus, three incidental durotomies, two episodes of symptomatic postoperative anemia, 11 unrelated traumas (motor vehicle accidents and non-inpatient falls), and 17 exacerbation of other medical conditions (e.g., anxiety, headaches, asthma).

**Table 1:** Characterization of adverse events. Adverse events listed and graded on expectancy, outcomes, severity, whether they were treated, and what the initial treatment was.

Variable	Response	Adverse events (N = 124)
Types of adverse event	Back pain	35 (28%)
	Radiculopathy	19 (15%)
	Back pain with radiculopathy	7 (6%)
	Wound infection	10 (8%)
	DVT/PE	6 (5%)
	Durotomy	3 (2%)
	Unrelated trauma	11 (9%)
	Infection not at the surgical site	9 (7%)
	Postoperative anemia requiring treatment	2 (1%)
	Constipation/ileus	5 (4%)
	Other medical issue	17 (14%)
Expectancy	Unexpected AE	40 (32%)
	Expected AE	84 (68%)
Outcome	Resolved	57 (46%)
	Unresolved	4 (3%)
	Ongoing at the end of the study	62 (50%)
	Death	1 (1%)
Severity	Mild	18 (15%)
	Moderate	64 (52%)
	Severe	26 (21%)
	Serious	16 (13%)
Treated	Yes	117 (95%)
	No	7 (5%)
Initial treatment	None	6 (5%)
	Observation, no action	8 (6%)
	Concomitant medication	58 (49%)
	Surgery/surgical intervention	8 (6%)
	Injections	30 (24%)
	Diagnostic testing	12 (10%)

Eighty-four (68%) AEs were expected, and 40 (32%) were unexpected. One patient died during the data collection period in relation to failure to thrive, 57 (46%) of the AEs had resolved, and 62 (50%) were ongoing at the end of the study. Almost all of the AEs were treated (n = 117, 95%). Initial treatments included observation (n = 8, 6%), concomitant medications (n = 58, 49%), surgery/surgical intervention (n = 8, 6%), diagnostic testing (n = 12, 10%), and other treatments (i.e., injections) (n = 30, 24%).

For the eight patients who underwent additional surgery, two underwent hematoma evacuation/wound exploration for postoperative weakness. One patient who originally underwent lumbar decompression for spinal stenosis required a cervical procedure two years later. Another patient underwent hip surgery for a femoral neck fracture 289 days after the spinal procedure. Two patients developed adjacent level disease an average of 2.5 years after surgery. One patient requested removal of hardware after a significant weight loss. One patient required revision of fusion for pseudoarthrosis 35 months after surgery.

Relationship of BMA with adverse events

None of the AEs were definitively related to the harvesting technique or the use of cBMA (Table 2). Seventy-six (61%) were clearly unrelated to the use of cBMA (e.g., UTI and exacerbation of medical comorbidities). The remaining 48 (39%) AEs (i.e., back pain and anemia) were possibly related to the use of cBMA.

**Table 2:** Relationship of adverse events to the use of cBMA.

Variable	Response	Adverse events (N = 124)
Relationship with cBMA	Unrelated	76 (61%)
	Possibly related to the use of cBMA	48 (39%)

Relationship of surgical procedure with adverse events

There were 67 AEs in 50 patients that were probably not related to surgical procedure (Table 3).

There were 35 AEs in 30 patients that were adverse reactions to surgical procedure. Wound infection (n = 8, 7%), deep vein thrombosis (DVT)/pulmonary embolism (PE) (n = 6, 5%), and constipation/ileus (n = 4, 2%), for example, were the most common types of AEs clearly related to having undergone surgery.

**Table 3:** Relationship of surgery to adverse events.

Variable	Response	Adverse events (N = 124)
Relationship with surgery	Probably not related	67 (54%)
	Possibly related	22 (18%)
	Definitely related	35 (28%)

Arthrodesis success

Follow-up imaging to allow for the assessment of fusion was available for 139 of the 150 patients (Table 4). There were 11 patients (five with AEs and six without AEs) without fusion data. Of the remaining 139 patients, 129 (92.8%) showed evidence of fusion. Of the patients with AEs, 38 (50%) had imaging demonstrating solid fusion within two years of their initial surgery (Table 5). Twenty-nine (38%) had evidence of fusion that was not yet completely spanning the disc space or facets at the surgical levels. Four (5%) patients had imaging demonstrating nonunion, and five (6.5%) did not have sufficient imaging for review. Similarly, in the 74 patients without AEs, 40 (54%) had imaging with evidence of solid fusion during follow-up, 22 (30%) had partial/progressing fusion, and six (8%) had evidence of nonunion. Only one patient required a subsequent surgical intervention for symptomatic nonunion.

Although the concentration of MSCs has previously been found to decline with age, failure of fusion was not associated with increased age in our study. The mean age of patients in the nonunion group (56.1 years) was not found to be statistically different from that of the successful fusion group (57.8 years) (p = 0.345).

**Table 4:** Arthrodesis success.

Variable	Response	Total number of patients (N = 150)
Success of fusion	Not available	11 (8%)
	Fusion	129 (86%)
	Pseudarthrosis	10 (6%)

**Table 5:** Relationship of adverse events to fusion success.

	Fusion	Nonunion	No data	Total
Adverse event	67	4	5	76
No adverse event	62	6	6	74
Total	129	10	11	

## Discussion

Clinical science research

The clinical use of BMA was originally supported by studies showing the benefit of MSCs in promoting and improving bony fusion [12]. Its current orthopedic applications have expanded, and it is being used to treat full-thickness cartilage lesions, osteochondral lesions, osteoarthritis, bone healing (nonunion or delayed union), and tendon repair [13].

The clinical literature investigating the use of BMA and MSCs in spine surgery consists mainly of small observational studies. The first clinical report of BMA being applied to spinal fusions was by Kitchel in 2006 [14]. He followed 25 patients with one-level posterior lumbar interbody fusion (PLIF) for 24 months, with a mineralized collagen bone graft substitute combined with BMA on one side and iliac crest autograft on the other (Table 6). The study showed similar arthrodesis rates, demonstrating that BMA may be an adequate substitute to the gold standard of iliac crest autograft. The ability of BMA and allograft to obtain fusions comparable with iliac crest autograft was confirmed in a subsequent study [15]. When combining BMA and demineralized bone matrix (DBM), an arthrodesis rate of 81.3% can be achieved [16]. The International Spine Study Group recently reported an 87% fusion rate in their retrospective review of anterior lumbar interbody fusions using mineralized collagen and BMA. However, this fusion rate is lower than the already reported robust anterior fusion rates of greater than 95% [17].

**Table 6:** Previously reported rates of BMA arthrodesis with concomitant biomaterials. DBM = demineralized bone matrix; BCP = biphasic calcium phosphates; B-TCP = beta-tricalcium phosphate.

	Authors (year)	No. of patients	Combined biomaterials	Arthrodesis rate
1	Kitchel (2006) [14]	25	Mineralized collagen	80%
2	Johnson (2014) [15]	24	Allograft, 1000 units of recombinant thrombin, and 100 mg of calcium chloride	80%
3	Ajiboye et al. (2016) [16]	80	DBM	81.3%
4	Hostin et al. (2016) [17]	22	Mineralized collagen	87%
5	Moro-Barrero et al. (2007) [18]	70	BCP	89%
6	Gan et al. (2008) [19]	41	B-TCP	95%
7	Bansal et al. (2009) [20]	30	BCP	100%
8	Niu et al. (2009) [21]	43	Laminectomy bone chips and calcium sulfate pellets	86%
9	Hart et al. (2014) [23]	30	Allograft	80%
10	Ajiboye et al. (2018) [26]	30	DBM	<65 years old: 76.4%; >65 years old: 36%
11	Piccirilli et al. (2017) [27]	11	Allograft	100%

Moro-Barrero et al. operated on 70 patients using autologous bone graft on the left side and biphasic calcium phosphate (BCP) ceramic mixed with BMA on the right side [18]. They found that the side with BMA had non-statistically significantly better fusion rates (89% versus 80%). Gan et al. followed by reporting a 95% fusion rate in 41 patients undergoing posterior spinal fusion with beta-tricalcium phosphate (B-TCP) and BMA [19]. Bansal et al. also published their results in 2009 using autograft on one side and a hydroxyapatite/B-TCP/BMA combination on the other side, showing 97% and 100% fusion rates, respectively [20]. Niu et al. conducted a similar study with laminectomy bone chips mixed with BMA and obtained 86% fusion rates compared with 90% on the control side [21]. Odri et al. investigated the concentrations of BMA and volumes of fusion masses. They saw no difference in fusion mass volumes in 15 patients using centrifuged BMA on one side with non-concentrated BMA on the other [22]. This was despite a sixfold increase in nucleated cells, a threefold increase in platelets, and a doubling of osteoprogenitor cells.

Perhaps the highest level of evidence on the use of BMA was a blinded, prospective, randomized control study. Hart et al. enrolled 80 patients receiving an instrumented posterolateral fusion [23]. One group received allograft chips alone (40% success rate), and the other group received allograft chips mixed with BMA (80% success rate), which illustrated definitively the ability of BMA to augment fusion.

It is important to note that the age of the patient from which the BMA is harvested could be an important factor. It is known that with senescence there is a decrease in the number of stem cells in the bone marrow, as well as their ability to differentiate into osteocytes [24]. In a study without a control arm in patients over 65 who were undergoing a transforaminal interbody fusion with the use of BMA and DBM, there was an 84% fusion rate at one-year follow-up [25]. When the same group added control arms, including patients under 65 years of age who received BMA/DBM and patients over 65 who received the gold standard of iliac crest bone graft, they found that BMA/DBM had a fusion rate of only 36% in patients over 65, compared with a 76.4% fusion rate in those under 65. Patients who received iliac crest bone grafts had a fusion rate of 80% in patients aged >65 [26].

One last note should be made regarding the use of adipose-derived MSCs. Piccirilli et al. supplemented their PLIFs in 11 patients with allograft/BMA and 11 patients with allograft/fat tissue-derived MSCs. Although all patients in the study reportedly fused, follow-up was limited to three months, and this was a single-center experience [27]. More studies are needed to come to a robust conclusion about the efficacy of BMA versus adipose-derived MSCs.

Limitations of BMA and MSCs

There are several limitations to the current literature regarding routine practical applications of BMA or MSCs to spine surgery. It is difficult to make direct comparisons in the basic science and animal model research of BMA and MSC in spine surgery because the data is heterogeneous; differences include which animal model was chosen, whether BMA or MSCs were applied to the model, how the MSCs were produced and cultured, the amount of MSCs applied, on which substrate they were applied, which anatomical section was fused, and how the fusion construct was identified. It is also important to note that bone healing in small animal models may not approximate human bone healing [28]. Without a standardized model with a standardized protocol, the translation of animal model research to clinical application will always have limitations.

Clinical human studies have similar difficulties. There are differences in the location where the BMA is harvested (iliac crest versus vertebral body), how the BMA is modified before implantation, whether autologous or allogeneic MSCs are implanted, how the MSCs are treated before implantation, how the BMA is applied, and the location of implantation. The viable optimal cell count that needs to be implanted is also debated, with mixed evidence that it matters. The age of the person from which BMA is aspirated is also a confounding factor, with evidence suggesting that the older the person, the less effective the MSCs [25]. There is also variability in the osteoconductive material used and the process of BMA/MSC expansion. In addition, more studies are needed to verify the quality of the bone produced with MSCs, including evaluations of mechanical strength and durability. Most importantly, a robust level I study accounting for the above variables is required to further demonstrate which materials and which protocol is efficacious in inducing arthrodesis.

Case series discussion

Our data found no definitive evidence of AEs in relation to the collection or use of cBMA. Certainly, general risks of spinal surgery (anemia, back pain, and blood loss) were noted and in line with published incidences. Our overall fusion success rate (92.8%) was consistent with previously reported studies [21]. The rate of fusion determined in this study is also similar to that found in studies analyzing fusion success with rhBMP-2 (92%) [29]. Age was not a significant factor in infusion success in our patient series, although, with the low pseudoarthrosis rate, it was not powered to detect a significant difference.

## Conclusions

In comparison to previous studies, which are generally small and observational in nature, our case series is comparatively more robust, with the analysis of 150 patients. Fusion was successful in 86% of the patients, pseudoarthrosis was present in 6%, and 11% did not have available postoperative fusion data. These rates of successful fusion are similar to the previous studies aforementioned. Interestingly, fusion failure was not associated with age in our study, despite the fact that stem cell availability decreases with senescence. Perhaps future studies that include a larger cohort of age-advanced patients could elucidate the clinical relevance of this physiology further.

In terms of adverse events, none could definitively be attributed to the harvesting technique or the use of BMA, and 61% of the adverse events were clearly unrelated (UTI and exacerbation of comorbidities). The remaining 39% of adverse events could have potentially been related, such as back pain (28%). However, it was difficult to elucidate whether this was synonymous with the common postoperative pain experienced after a lumbar fusion, as opposed to pain from the graft harvesting itself. Overall, there did not appear to be a significant increase in adverse events compared to non-BMA harvested lumbar fusion procedures. Perhaps even more importantly, the occurrence of an adverse event related to surgery had no negative impact on the likelihood of fusion success. 

Overall, our study and those previous to it demonstrate that the use of BMA offers the benefits of autologous MSCs and endogenous trophic factors without the risks incurred during the open surgical harvesting of an iliac crest graft. From our findings, it can be concluded that the use of BMA is at least as effective as other adjuncts for improving the success rates of fusion and is thus a viable operative option. In terms of future research, it may be helpful to determine if fusion success varies by specific surgical procedure performed (e.g., stratifying success rates by posterior lumbar interbody fusion versus transforaminal lumbar interbody fusion). As in previous studies, our method involved the combination of cBMA with other biomaterials to promote fusion. Future studies could assess success rates in the cohorts of patients with varying biomaterial combination profiles (e.g., cBMA in conjunction with allograft versus autograft versus DBM versus BCP).

## References

[1] Mroz TE, Wang JC, Hashimoto R, Norvell DC (2010). Complications related to osteobiologics use in spine surgery: a systematic review. Spine (Phila Pa 1976).

[2] Kim DH, Rhim R, Li L (2009). Prospective study of iliac crest bone graft harvest site pain and morbidity. Spine J.

[3] Gruskay JA, Basques BA, Bohl DD, Webb ML, Grauer JN (2014). Short-term adverse events, length of stay, and readmission after iliac crest bone graft for spinal fusion. Spine (Phila Pa 1976).

[4] Tuchman A, Brodke DS, Youssef JA (2017). Autograft versus allograft for cervical spinal fusion: a systematic review. Global Spine J.

[5] Hisenkamp M, Muyelle L, Eastlund T, Fehily D, Noel L, Strong DM (2012). Adverse reaction and events related to musculoskeletal allografts: reviewed by the World Health Organisation Project NOTIFY. Int Orthop.

[6] Carragee EJ, Baker RM, Benzel EC (2012). A biologic without guidelines: the YODA project and the future of bone morphogenetic protein-2 research. Spine J.

[7] James AW, Lachaud G, Shen J (2016). A review of the clinical side effects of bone morphogenic protein-2. Tissue Eng Part B Rev.

[8] McAnany SJ, Ahn J, Elboghdady IM (2016). Mesenchymal stem cell allograft as a fusion adjunct in one- and two-level anterior cervical discectomy and fusion: a matched cohort analysis. Spine J.

[9] Kelly MP, Lenke LG, Bridwell KH, Agarwal R, Godzik J, Koester L (2013). Fate of the adult revision spinal deformity patient: a single institution experience. Spine (Phila Pa 1976).

[10] Cavagna R, Tournier C, Aunoble S, Bouler JM, Antonietti P, Ronai M, Le Huec JC (2008). Lumbar decompression and fusion in elderly osteoporotic patients: a prospective study using less rigid titanium rod fixation. J Spinal Disord Tech.

[11] Chahla J, Mannava S, Cinque ME, Geeslin AG, Codina D, LaPrade RF (2017). Bone marrow aspirate concentrate harvesting and processing technique. Arthrosc Tech.

[12] Hernigou P, Poignard A, Beaujean F, Rouard H (2005). Percutaneous autologous bone-marrow grafting for nonunions. Influence of the number and concentration of progenitor cells. J Bone Joint Surg Am.

[13] Gianakos AL, Sun L, Patel JN, Adams DM, Liporace FA (2017). Clinical application of concentrated bone marrow aspirate in orthopaedics: a systematic review. World J Orthop.

[14] Kitchel SH (2006). A preliminary comparative study of radiographic results using mineralized collagen and bone marrow aspirate versus autologous bone in the same patients undergoing posterior lumbar interbody fusion with instrumented posterolateral lumbar fusion. Spine J.

[15] Johnson RG (2014). Bone marrow concentrate with allograft equivalent to autograft in lumbar fusions. Spine (Phila Pa 1976).

[16] Ajiboye RM, Eckardt MA, Hamamoto JT, Plotkin B, Daubs MD, Wang JC (2016). Outcomes of demineralized bone matrix enriched with concentrated bone marrow aspirate in lumbar fusion. Int J Spine Surg.

[17] Hostin R, O'Brien M, McCarthy I, Bess S, Gupta M, Klineberg E, International Spine Study Group DCO (2016). Retrospective study of anterior interbody fusion rates and patient outcomes of using mineralized collagen and bone marrow aspirate in multilevel adult spinal deformity surgery. Clin Spine Surg.

[18] Moro-Barrero L, Acebal-Cortina G, Suarez-Suarez M, Perez-Redondo J, Murcia-Mazon A, Lopez-Muniz A (2007). Radiographic analysis of fusion mass using fresh autologous bone marrow with ceramic composites as an alternative to autologous bone graft. J Spinal Disord Tech.

[19] Gan Y, Dai K, Zhang P, Tang T, Zhu Z, Lu J (2008). The clinical use of enriched bone marrow stem cells combined with porous beta-tricalcium phosphate in posterior spinal fusion. Biomaterials.

[20] Bansal S, Chauhan V, Sharma S, Maheshwari R, Juyal A, Raghuvanshi S (2009). Evaluation of hydroxyapatite and beta-tricalcium phosphate mixed with bone marrow aspirate as a bone graft substitute for posterolateral spinal fusion. Indian J Orthop.

[21] Niu CC, Tsai TT, Fu TS, Lai PL, Chen LH, Chen WJ (2009). A comparison of posterolateral lumbar fusion comparing autograft, autogenous laminectomy bone with bone marrow aspirate, and calcium sulphate with bone marrow aspirate: a prospective randomized study. Spine (Phila Pa 1976).

[22] Odri GA, Hami A, Pomero V (2012). Development of a per-operative procedure for concentrated bone marrow adjunction in postero-lateral lumbar fusion: radiological, biological and clinical assessment. Eur Spine J.

[23] Hart R, Komzak M, Okal F, Nahlik D, Jajtner P, Puskeiler M (2014). Allograft alone versus allograft with bone marrow concentrate for the healing of the instrumented posterolateral lumbar fusion. Spine J.

[24] Zhou S, Greenberger JS, Epperly MW, Goff JP, Adler C, Leboff MS, Glowacki J (2008). Age-related intrinsic changes in human bone-marrow-derived mesenchymal stem cells and their differentiation to osteoblasts. Aging Cell.

[25] Ajiboye RM, Hamamoto JT, Eckardt MA, Wang JC (2015). Clinical and radiographic outcomes of concentrated bone marrow aspirate with allograft and demineralized bone matrix for posterolateral and interbody lumbar fusion in elderly patients. Eur Spine J.

[26] Ajiboye RM, Eckardt MA, Hamamoto JT, Sharma A, Khan AZ, Wang JC (2018). Does age influence the efficacy of demineralized bone matrix enriched with concentrated bone marrow aspirate in lumbar fusions?. Clin Spine Surg.

[27] Piccirilli M, Delfinis CP, Santoro A, Salvati M (2017). Mesenchymal stem cells in lumbar spine surgery: a single institution experience about red bone marrow and fat tissue derived MSCs. J Neurosurg Sci.

[28] Aspenberg P, Wang E, Thorngren KG (1992). Bone morphogenetic protein induces bone in the squirrel monkey, but bone matrix does not. Acta Orthop Scand.

[29] Lee DD, Kim JY (2017). A comparison of radiographic and clinical outcomes of anterior lumbar interbody fusion performed with either a cellular bone allograft containing multipotent adult progenitor cells or recombinant human bone morphogenetic protein-2. J Orthoped Surg Res.

